# Гонадотропинзависимое преждевременное половое развитие: молекулярно-генетические и клинические характеристики

**DOI:** 10.14341/probl13215

**Published:** 2023-05-11

**Authors:** Д. А. Хабибуллина, А. А. Колодкина, Т. В. Визеров, Н. А. Зубкова, О. Б. Безлепкина

**Affiliations:** Национальный медицинский исследовательский центр эндокринологии; Национальный медицинский исследовательский центр эндокринологии; Национальный медицинский исследовательский центр эндокринологии; Национальный медицинский исследовательский центр эндокринологии; Национальный медицинский исследовательский центр эндокринологии

**Keywords:** гонадотропинзависимое преждевременное половое развитие, наследственные формы, MKRN3, молекулярногенетический анализ, семейный анамнез

## Abstract

**ОБОСНОВАНИЕ:**

ОБОСНОВАНИЕ. В 90% случаев среди девочек и до 25–60% среди мальчиков генез гонадотропинзависимого преждевременного полового развития (ППР) остается неясным. Известно, что 25–27,5% случаев гонадотропинзависимого ППР являются моногенными вариантами и предполагают аутосомно-доминантный характер наследования с неполной пенетрантностью, зависящей от пола. В настоящее время патогенные варианты в генах KISS1, KISS1R, MKRN3, DLK1 ассоциированы с преждевременной активацией гипоталамо-гипофизарно-гонадной оси в детстве. Генетическая верификация диагноза у пациентов с наследственными формами ППР позволяет расширить наши представления о природе патологии и крайне необходима для проведения медико-генетического консультирования.

**ЦЕЛЬ:**

ЦЕЛЬ. Изучение клинических особенностей и молекулярно-генетических характеристик пациентов с идиопатическим гонадотропинзависимым ППР.

**МАТЕРИАЛЫ И МЕТОДЫ:**

МАТЕРИАЛЫ И МЕТОДЫ. Обследована группа пациентов с идиопатическим гонадотропинзависимым ППР, наследственный анамнез которых отягощен по раннему и/ или преждевременному половому созреванию. Всем пациентам проведено комплексное обследование, включая лабораторно-инструментальные методы диагностики и полноэкзомное секвенирование методом NGS (next-generation sequencing).

**РЕЗУЛЬТАТЫ:**

РЕЗУЛЬТАТЫ. В исследование включены 30 пациентов (29 девочек, 1 мальчик) с идиопатическим гонадотропинзависимым ППР. Медиана возраста пациентов на момент обследования составила 7,2 года [6,5; 7,7]. Во всех случаях имел место отягощенный семейный анамнез: в 40% случаев — по отцовской линии, в 37% случаев — по материнской линии, в 23% случаев ППР диагностировано у сибсов. Полноэкзомное секвенирование проведено 21 пациенту: в 61,9% случаев (95% ДИ [40; 79]) идентифицированы изменения нуклеотидной последовательности в генах-кандидатах, ассоциированных с гонадотропинзависимым ППР. В подавляющем большинстве (у 77% пациентов) выявлялся дефект гена MKRN3 (95% ДИ [49; 92], что согласуется с зарубежными данными о его наибольшей распространенности в структуре моногенных форм ППР, в 23% случаев (95% ДИ [7; 50]) — в других генах-кандидатах, ассоциированных с нейроонтогенезом и нейроэндокринными механизмами регуляции гипоталамо-гипофизарной оси.

**ЗАКЛЮЧЕНИЕ:**

ЗАКЛЮЧЕНИЕ. Проведенное исследование демонстрирует важность детального сбора наследственного анамнеза у детей с ППР для определения показаний к проведению молекулярно-генетического анализа. Наличие данных о характере наследования и клинических проявлениях моногенных форм ППР позволит упростить диагностику наследственных форм заболевания, проводить медико-генетическое консультирование семей с последующим своевременным обследованием и назначением патогенетической терапии заболевшим.

## Abstract (en)

**BACKGROUND:**

BACKGROUND: In 90% cases of girls and 25–60% cases of boys the cause of gonadotropin-dependent precocious puberty (PP) is unclear. Up to 25–27.5% of gonadotropin-dependent PP cases are monogenic and suggest autosomal-dominant inheritance with incomplete sex-dependent penetrance. To date, mutations in genes KISS1, KISS1R, MKRN3, DLK1 have been described as causal variants leading to precocious hypothalamic-pituitary axis activation in childhood. Genetic testing in patients with hereditary forms of PP can expand our knowledge of underlying molecular mechanisms of the disease and it  is also necessary for genetic counselling.

**AIM:**

AIM: To study clinical features and genetic characteristics of patients with idiopathic gonadotropin-dependent precocious puberty.

**MATERIALS AND METHODS:**

MATERIALS AND METHODS: A group of patients with idiopathic gonadotropin-dependent precocious puberty and positive family history (early or precocious puberty) was examined. Laboratory and instrumental diagnostic tests, full-exome sequencing (NGS, next-generation sequencing) were provided for all patients.

**RESULTS:**

RESULTS: The study included 30 patients (29 girls, 1 boy) with idiopathic gonadotropin-dependent precocious puberty. The median of patients age at the time of the examination was 7,2 years [6,5; 7,7]. Positive family history presented in all cases: in 40% of patients on father’s side, in 37% — on mother’s side, in 23% of patients PP was diagnosed in siblings. The fullexome sequencing was conducted to 21 patients: in 61,9% of cases (95% CI [40;79]) nucleotide variants were identified   in genes, associated with gonadotropin-dependent precocious puberty. MKRN3 gene defect was detected in most cases (77% cases (95% CI [49; 92]), which consistent with international data on its highest prevalence in the monogenic forms of PP. In 23% of cases (95% CI [7; 50]) nucleotide variants were identified in other candidate genes associated with neuroontogenesis and neuroendocrine regulation mechanisms of hypothalamic-pituitary axis.

**CONCLUSION:**

CONCLUSION: Our study confirms that detailed family history data in children with PP provides a rational approach to molecular-genetic testing. Data of inheritance pattern and clinical manifestations will simplify the diagnosis of hereditary forms of disease and enhance genetic counselling of families, followed by timely examination and administration of pathogenetic therapy.

## ОБОСНОВАНИЕ

Центральное (гонадотропинзависимое) преждевременное половое развитие (ППР) представляет собой симптомокомплекс, обусловленный преждевременной активацией механизма импульсной секреции гонадотропин-рилизинг-гормона (ГнРГ) вследствие функциональных и/или органических поражений ЦНС, что приводит к преждевременному высвобождению лютеинизирующего (ЛГ) и фолликулостимулирующего (ФСГ) гормонов [1–4]. Согласно российским клиническим рекомендациям, половое созревание расценивается как преждевременное при появлении вторичных половых признаков у девочек в возрасте до 8 лет и у мальчиков в возрасте до 9 лет [1]. По данным мировой литературы, частота встречаемости гонадотропинзависимого ППР составляет от 1:5000 до 1:10 000 детского населения, значительно чаще встречаясь среди девочек. В 90% случаев среди девочек и 25–60% среди мальчиков генез преждевременной активации гипоталамо-гипофизарно-гонадной оси (ГГГО) остается неясным, и, в случае исключения органической патологии центральной нервной системы, обозначается как идиопатическое [5–7] .

В настоящее время ведется множество научных исследований, предметом которых является изучение механизмов, регулирующих активность ГГГО. Доказательства генетической регуляции являются неоспоримыми, что подтверждено рядом популяционных исследований, в которых продемонстрирована корреляция между возрастом начала полового созревания у детей и их родителей, а также более высокая согласованность сроков развития вторичных половых признаков, включая менархе, у монозиготных близнецов. Кроме того, до 27,5% случаев центрального преждевременного полового созревания являются моногенными вариантами и предполагают аутосомно-доминантный характер наследования с неполной пенетрантностью, зависящей от пола [8–11].

В последнее десятилетие было идентифицировано несколько десятков генов в сети нейроэндокринных факторов, которые непосредственно модулируют сроки полового созревания. Несмотря на то что установить молекулярно-генетическую основу заболевания удается не всегда, патогенные варианты в генах KISS1, KISS1R, MKRN3, DLK1 в настоящее время ассоциированы с преждевременной активацией ГГГО в периоде детства [11–14].

В 2008 г. впервые был обнаружен ген, активирующие мутации в котором являются причиной преждевременной активации ГГГО — KISS1R, ранее известный как GPR54. Активирующая мутация гена рецептора кисспептина была идентифицирована у девочки с идиопатическим гонадотропинзависимым ППР. Исследования in vitro показали, что наличие мутации приводит к длительной активации внутриклеточных сигнальных путей после связывания кисспептина с рецептором, что может усиливать стимулирующие эффекты лиганда на секрецию GnRH, ускоряя тем самым созревание репродуктивной оси [12][15]. Дальнейшие исследования позволили предположить, что мутации гена кисспептина (KISS1) опосредуют большую стабильность мутантного нейропептида и его устойчивость к деградации. Подобные изменения приводят к большей способности стимулировать передачу сигнала к рецептору, являясь причиной преждевременного полового созревания [13].

Роль MKRN3 в патогенезе гонадотропинзависимых форм ППР впервые была продемонстрирована А. Abreu и соавт. в 2013 г., когда по данным полноэкзомного секвенирования были идентифицированы 15 носителей мутаций с классическими проявлениями центрального ППР [11]. Полученные результаты послужили поводом для дальнейшего поиска мутаций в данном гене у пациентов с семейными формами гонадотропинзависимого ППР, и к настоящему времени описано около 30 мутаций в кодирующих и промоторной областях гена [16–18]. Cуществует гипотеза о супрессивном влиянии MKRN3 на активность ГнРГ-секретирующих нейронов, в частности KISS1 нейронов, как ключевых стимуляторов секреции ГнРГ. Данные подтверждены исследованиями in vivo, в которых продемонстрировано прогрессирующее снижение экспрессии MKRN3 в гипоталамусе грызунов и приматов по мере прогрессии полового созревания. Таким образом был сделан вывод об ингибирующем влиянии гена в периоде пубертата, тогда как потеря его функции способствует преждевременной стимуляции секреции ГнРГ и старту полового созревания. [7][19][20]. По данным многочисленных зарубежных исследований мутации в гене MKRN3 считаются наиболее частой причиной моногенных случаев ППР, составляя до 46% среди семейных вариантов и около 4% — среди спорадических случаев [8][11][21][22].

В 2017 г. А. Dauber и соавт. опубликовали исследование с описанием не синдромальной формы ППР, где у пяти членов одной семьи идентифицирован патогенный вариант в гене DLK1 [14]. Последующие исследования позволили сделать предположение, что высокая экспрессия DLK1 в гипоталамусе опосредует стимулирующее влияние кисспептиновых нейронов на секрецию ГнРГ [14][23]. Несмотря на небольшую частоту выявления патогенных вариантов гена, клиническая картина при инактивирующих мутациях DLK1 характеризуется специфическим фенотипом, включая раннюю манифестацию избыточного веса и/или ожирения, нарушения липидного и углеводного обменов.

Однако, в большинстве случаев причина ППР остается невыясненной, что требует дальнейших исследований. Генетическая верификация диагноза у пациентов с наследственными формами ППР необходима для проведения медико-генетического консультирования, а также позволяет расширить наши представления о молекулярно-генетической природе патологии. Ранняя клиническая диагностика и своевременная терапия крайне важны, так как преждевременное половое созревание и менархе связаны с неудовлетворительным ростовым прогнозом и психосоциальной дезадаптацией, а также с повышенным риском метаболических нарушений, сердечно-сосудистых заболеваний, злокачественных новообразований [8][24].

## ЦЕЛЬ ИССЛЕДОВАНИЯ

Изучить клинические особенности, гормонально-метаболические и молекулярно-генетические характеристики пациентов с идиопатическим гонадотропинзависимым ППР.

## МАТЕРИАЛЫ И МЕТОДЫ

Место и время проведения исследования

Место проведения. Обследование пациентов проведено в Институте детской эндокринологии ФГБУ «НМИЦ эндокринологии» Минздрава России

Время исследования. В исследование включены пациенты, обследованные Институте детской эндокринологии ФГБУ «НМИЦ эндокринологии» Минздрава России с 2019 по 2022 гг.

Изучаемые популяции (одна или несколько)

Популяция: 30 пациентов с идиопатическим гонадотропинзависимым ППР, наследственный анамнез которых отягощен по раннему и/ или преждевременному половому созреванию.

Критерии включения: манифестация полового созревания у девочек в возрасте до 8 лет, у мальчиков в возрасте до 9 лет, наличие отягощенного семейного анамнеза по раннему и/ или ППР (по материнской и/ или отцовской линии), а также письменное информированное согласие родителей или законного опекуна пациента об участии в исследовании

Критерии невключения: наличие органической патологии ЦНС.

Способ формирования выборки из изучаемой популяции: сплошной.

Дизайн исследования

Данная работа представляет собой одноцентровое интервенционное одномоментное одновыборочное несравнительное исследование, включившее 30 пациентов с гонадотропинзависимым ППР при наличии отягощенного семейного анамнеза. Набор пациентов проводился на основании соответствия критериям включения и отсутствия критериев исключения. Всем пациентам проведено комплексное обследования, включавшее стандартные и специальные методы.

Описание медицинского вмешательства (для интервенционных исследований)

Протокол исследования включал в себя клиническое обследование пациентов с подробным сбором наследственного анамнеза, с физикальным осмотром с оценкой фенотипических особенностей, антропометрических показателей (расчет SDS роста, скорости роста, целевого и конечного прогнозируемого роста по Bayley-Pinneau проведен с помощью компьютерной программы Auxology 1,0 (Pfizer, США) и оценкой стадии полового развития по шкале Таннер).

Лабораторная диагностика включала оценку уровней ЛГ, ФСГ, эстрадиола у девочек и тестостерона у мальчиков.

Инструментальная диагностика включала проведение УЗИ органов малого таза у девочек и органов мошонки у мальчиков, рентгенографию кистей рук с оценкой костного возраста, МРТ головного мозга.

Лабораторное исследование уровней ЛГ, ФСГ, эстрадиола и тестостерона проводилось на автоматическом иммунохемилюминесцентном анализаторе Vitros 3600 (Ortho Clinical Diagnostics, «Johnson& Johnson», США) в клинико-диагностической лаборатории ФГБУ «НМИЦ эндокринологии» Минздрава России. За допубертатные нормы половых стероидов принимались уровни ЛГ менее 0,2 Ед/л, эстрадиола менее 55 пмоль/л, тестостерона менее 0,6 нмоль/л [1][ 2][25]. Функциональная проба с аналогом ГнРГ (Бусерелин 150 мкг интраназально) проведена всем пациентам кроме 4 девочек, у которых наступило менархе (оценивались базальные и стимулированные уровни ЛГ и ФСГ на 60 и 240 минутах).

Ультразвуковое исследование проводилось в консультативно-диагностическом центре ФГБУ «НМИЦ эндокринологии» Минздрава России на ультразвуковом сканере (Voluson E8, GE Healthcare, Австрия) с использованием конвексного датчика с частотой 3,5–5 Мгц при проведении УЗИ органов малого таза и с использованием линейного датчика с частотой 1–12 Мгц при проведении УЗИ органов мошонки. За норму для девочек допубертатного возраста (2–7 лет) принимались размеры матки соответствующие 3,2х1,5х0,9 см, а объем яичников не превышающий 1,7 см3 [1][25].

Рентгенография кистей с лучезапястными суставами проводилась в прямой проекции по стандартной методике с оценкой костного возраста по рентгенологическому атласу Tanner Whitehouse (TW-20).

Магнитно-резонансная томография головы проводилось на аппарате Optima MR450w, (GE Healthcare) c напряженностью магнитного поля 1,5 Тесла. Исследование проводилось в Т1 и Т2 взвешенных режимах по стандартной методике.

Молекулярно-генетический анализ проводился в лаборатории генетики моногенных эндокринных заболеваний Института персонализированной медицины ФГБУ «НМИЦ эндокринологии» Минздрава России методом массового параллельного секвенирования (next-generation sequencing, NGS) на платформе Illumina методом парно-концевого чтения (2x100 п.о.). Забор крови производился из локтевой вены вне зависимости от приема пищи в пробирки с консервантом этилендиаминтетраацетатом в концентрации 1,2–2,0 мг на 1 мл крови. Геномную ДНК извлекали роботизированной станцией Allsheng Autopure-96 (Hangzhou Allsheng Instruments Co., Ltd., China) из периферической крови с использованием набора для выделения геномной ДНК из цельной крови NucleoMag Blood (MN). Выделенную ДНК качественно и количественно анализировали с помощью спектрофотометра Eppendorf Biospectrometer Fluorescence (Eppendorf AG, Germany) и набора Qubit dsDNA HS Assay (Invitrogen, Carlsbad, CA, USA) соответственно.

Подготовку полногеномной библиотеки (KAPA HyperPlus, Roche, Швейцария) и обогащение матрицы ДНК (KAPA HyperCapture, Roche, Швейцария) производили в соответствии с протоколами производителя используя набор зондов KAPA HyperExome (Roche, Швейцария).

Обработка данных секвенирования проведена с использованием автоматизированного алгоритма, включающего выравнивание прочтений на референсную последовательность генома человека (HG38), постпроцессинг выравнивания, выявление вариантов и фильтрацию вариантов по качеству, а также аннотацию выявленных вариантов по всем известным транскриптам каждого гена из базы RefSeq с применением компьютерных алгоритмов предсказания патогенности вариантов (SIFT, PolyPhen-2 HDIV, Polyphen-2 HVAR, PROVEAN, CADD). Для оценки популяционных частот выявленных вариантов использованы данные международного проекта gnomAD Exomes для экзонных вариантов и базы gnomAD Genomes для интронных вариантов. Для предсказания эффекта изменений в сайтах сплайсинга и прилежащих к сайту сплайсинга интронных участках использованы программы SpliceAI и AdaBoost. Для оценки клинической релевантности выявленных вариантов использованы база данных OMIM, HGMD, специализированные базы данных по отдельным заболеваниям (при наличии) и литературные данные. Заключение о клинической значимости найденных вариантов дано с учетом рекомендаций American College of Medical Genetics and Genomics (ACMG) и российского руководства по интерпретации данных NGS. В заключение включены только варианты, имеющие возможное отношение к клиническим проявлениям у пациента. Полиморфизмы, классифицированные по различным критериям как нейтральные, не включены в заключение. Средняя глубина покрытия была не менее 70x, процент целевых нуклеотидов с эффективным покрытием >10х — не менее 97%.

Статистический анализ

Размер выборки предварительно не рассчитывался. Обработка и анализ статистических данных проводился в программах Statistica 8.0 (StatSoft, США), MS Exel 2010 (Microsoft, США). Количественные данные представлены в виде среднего, минимального и максимального значений (мин; макс) для ультразвуковых размеров матки и яичников у девочек, и медианы и интерквартильного размаха остальных показателей: Ме [Q1; Q3]. Качественные данные представлены в виде частот (%), 95-процентный доверительный интервал (ДИ) для относительных частот рассчитан с помощью метода Клоппера–Пирсона.

Этическая экспертиза

Исследование одобрено локальным этическим комитетом ФГБУ «НМИЦ эндокринологии» Минздрава России, протокол № 26 от 22.12.2021. Информированное согласие получено от родителей и/или законных опекунов всех обследованных пациентов.

## РЕЗУЛЬТАТЫ

В исследование включены 30 пациентов (29 девочек, 1 мальчик) с идиопатическим ППР центрального генеза. Наследственный анамнез у всех пациентов отягощен по раннему и/или ППР: у 12 пациентов (40%) отягощенный анамнез установлен по отцовской линии, у 11 пациентов (37%) — по материнской линии, в 7 случаях ППР отмечалось у сибсов (23%). Медиана возраста пациентов на момент обследования составляла 7,2 года [ 6,5; 7,7].

Первым симптомом у девочек являлось телархе, медиана возраста манифестации — 6 лет [ 5,0; 6,6]. На момент обследования в Институте детской эндокринологии 64,3% девочек имели II стадию развития молочных желез по шкале Таннер (B2), 35,7% девочек — III стадию (B3). В 60,7% случаев лобковое оволосение соответствовало I стадии по шкале Таннер (Р1), в 39,3% — II стадии (Р2). У четырех пациенток (14,2%) на момент обследования имелось менархе: медиана наступления менархе — 8,1 лет [ 7,2; 8,9]. У единственного мальчика первые признаки заболевания отмечены в 8,1 лет, а на момент обследования половое развитие соответствовало III стадии по шкале Таннер, объем яичек — 10 мл (d=s).

У всех пациентов отмечалось ускорение темпов роста и костного созревания: медиана SDS роста составила 1,4  [ 1,9; 2,5], медиана скорости роста — 8 см в год [ 8,4; 9,3], медиана SDS скорости роста составила +2,0 [ 2,9;3,4], медиана костного возраста — 8,8 лет [ 9,8; 11]. Медиана опережения костного возраста относительно паспортного составила 1,9 лет [ 2,7; 1,4].

Базальный уровень ЛГ превышал допубертатные значения у 96,5% пациентов: медиана ЛГ — 0,9 Ед/л [ 1,5; 4,0], а уровни половых стероидов у 100% пациентов (медиана эстрадиола 73,5 пмоль/л [ 86,8; 142], уровень тестостерона у мальчика составил 5,9 нмоль/л. Результаты стимуляционной пробы с бусерелином подтвердили гонадотропинзависимый генез ППР: медиана максимального уровня ЛГ составила 24,2 Ед/л [ 24,2; 40,0], ФСГ — 22,5 Ед/л [ 13,1; 31,7]. Ни в одном из случаев не отмечено превышения стимулированных уровней ФСГ над ЛГ, характерного для изолированного телархе.

По результатам ультразвукового исследования органов малого таза у девочек размеры матки превышали возрастные допубертатные нормы: среднее значение длины матки составляло — 3,32 см (2,2; 5), ширины матки — 2,2 см (1,3; 4,3), толщины матки –1,6 см (0,9; 2,8). Средний объем правого яичника составил 3,2 см3 (0,9; 9,2), левого яичника — 3 см3 (0,6; 7,2], что также превышало допубертатные нормы.

Принимая во внимание данные наследственного анамнеза 21 пациенту проведено молекулярно-генетическое исследование методом массового параллельного секвенирования (next-generation sequencing, NGS). Изменения нуклеотидной последовательности в генах — кандидатах, ассоциированных с гонадотропинзависимым ППР обнаружены у 13 пациентов (61,9% случаев, 95% ДИ [ 40; 79].

В 77% случаев (95% ДИ [ 49; 92]) идентифицированы варианты в гене MKRN3, в 23% случаев (95% ДИ [ 7; 50]) — в других генах-кандидатах, ассоциированных с ППР: MAPK8IP3 (omim no. 605431), POU1F1 (omim no. 173110) и NPFF1R (omin no. 607448) (табл. 1).

**Table:** Таблица 1. Результаты молекулярно-генетического анализа и некоторые клинические характеристики пациентов Примечание. МЛ — материнская линия, ОЛ — отцовская линия, С — сибс, П — патогенный, ВП — вероятно патогенный, Н — нейтральный, НКЗ — неопределенная клиническая значимость, M — missense, N — nonsense, Fs — frameshift, ASMG — American College of Medical Genetics and Genomics, In silico анализ — биоинформатические алгоритмы предсказательности патогенности.

№ пациента	Возраст манифестации ППР	Возраст обследования	Стадия полового развития по Таннер	Семейный анамнез по ППР	Молекулярно-генетическое исследование
Ген	Вариант, белок	Тип мутации	In silico анализ	Патогенность ACMG
1	4 года	7,5 лет	В2 Р2	МЛ	MAPK8IP3 (NM_001318852.2)	c.3830G>T (p.Gly1277Val)	M	Н	НКЗ
2	4 мес.	4,1 лет	В2 Р1	МЛ	POU1F1 (NM_000306.4)	c.370A>G (p.Met124Val)	M	Н	НКЗ
3	7,2 лет	7,4 лет	В2 Р1	МЛ	NPFFR1 (NM_022146.5)	c.452A>T (p.Glu151Val)	M	Н	НКЗ
4	5 лет	6,6 лет	В3 Р1	ОЛ	MKRN3 (NM_005664.4)	c.690dupT (p.T230fs)	Fs	П	ВП
5	6 лет	7,8 лет	В2 Р1	ОЛ	MKRN3 (NM_005664.4)	c.1091G>C (p.Cys364Ser)	M	П	ВП
6	4,2 лет	5,1 лет	В2 Р1	ОЛ, С	MKRN3 (NM_005664.4)	с.118G>T (р.E40X)	N	П	ВП
7	5 лет	9,1 лет	В4 Р4, Менархе+	ОЛ, С	MKRN3 (NM_005664.4)	с.118G>T (р.E40X)	N	П	ВП
8	6,5 лет	6,11 лет	В2 Р1	С	MKRN3 (NM_005664.4)	c.343T>A (p.Cys115Ser)	M	П	НКЗ
9	5 лет	6,9 лет	В2 Р1	С	MKRN3 (NM_005664.4)	c.343T>A (p.Cys115Ser)	M	П	НКЗ
10	6,1 лет	7 лет	В2 Р1	ОЛ	MKRN3 (NM_005664.4)	c.1091G>C, (p.Cys364Ser)	M	П	ВП
11	8 лет	8,3 лет	В3 Р1, Менархе+	С	MKRN3 (NM_005664.4)	c.1088A>G, (p.Gln363Arg)	M	ВП	НКЗ
12	7,7 лет	8 лет	В3 Р1	С	MKRN3 (NM_005664.4)	c.1088A>G, (p.Gln363Arg)	M	ВП	НКЗ
13	6 лет	7,3 лет	В3 Р2, Менархе+	ОЛ	MKRN3 (NM_005664.4)	c.1199G>C, (p.Cys400Ser)	M	П	ВП

## ОБСУЖДЕНИЕ

По данным зарубежных авторов, около 27,5% семейных случаев ППР являются моногенными [11]. В нашем исследовании вариантные замены были выявлены в 61,9% случаев. В подавляющем большинстве выявлялся дефект гена MKRN3 (77–95% ДИ [49; 92], что подтверждает его наибольшую распространенность в структуре моногенных форм ППР [8][9][11].

В проведенном нами исследовании клиническая картина наследственных, в том числе моногенных, форм ППР сопоставима с иными центральными формами заболевания, характеризуясь последовательным развитием вторичных половых признаков в сочетании с ускорением темпов роста и костного созревания. Средний возраст манифестации заболевания среди девочек соответствовал 6 годам [ 5,0;6,6], что сопоставимо с зарубежными данными [10][26][27]. Возраст начала пубертата у мальчика в нашем исследовании соответствовал 8,5 годам.

В исследовании L. Valadares и соавт., включавшем 880 пациентов с идиопатическим ППР, у 89 из которых описаны патогенные варианты MKRN3, специфичных лабораторно-инструментальных данных не выявлено: медианы базального и стимулированного уровней ЛГ составляли 1,27 МЕ/л и 22,0 МЕ/л соответственно, медиана ускорения костного возраста соответствовала 2,3 годам [16], что согласуется с результатами нашего исследования. В 2021 г. Seraphim и соавт. также сообщили об отсутствии отличительных диагностических показателей среди 71 пациента с мутациями MKRN3.

В нашей когорте патогенные варианты обнаружены только среди девочек, что отличается от международных данных о наибольшей распространенности мутаций MKRN3 среди мальчиков [16][18], однако учитывая ограничения исследуемой нами группы (недостаточное число зарегистрированных мальчиков) достоверно проанализировать данный показатель не представилось возможным.

Ген MKRN3, локализованный на длинном плече 15 хромосомы в критической области синдрома Прадера-Вилли (15q11.2), характеризуется импринтинговым характером наследования. Отсутствие экспрессии материнского аллеля определяет специфичный тип наследования — заболевание развивается в случаях, когда дефект унаследован по отцовской линии [7][19]. Заподозрить дефект данного гена возможно при детальном сборе семейного анамнеза, анализируя полученные данные в контексте возможного импринтингового типа наследования. В нашей когорте у 5 пациенток достоверно прослеживался характерный семейный анамнез по отцовской линии, что позволило идентифицировать моногенный генез заболевания. Однако, неполная пенетрантность гена позволяет объяснить отсутствие характерных наследственных данных у другой части пациентов или же свидетельствовать в пользу спорадического характера заболевания. В литературе также имеются сообщения о бессимптомном носительстве патогенных вариантов, подтверждая вероятность неполной пенетрантности гена [28][29].

В значительно меньшем количестве (23% случаев, 95% ДИ [ 7; 50]) в нашем исследовании удалось идентифицировать ранее не описанные варианты нуклеотидных последовательностей в других генах — кандидатах, ассоциированных с центральным ППР. Поводом для проведения генетического тестирования стал подробный анализ наследственного анамнеза: у двух пациенток раннее половое созревание с преждевременным менархе прослеживалась по материнской линии, в другом случае ППР диагностировано у единоутробной сестры.

Первой находкой стала гетерозиготная замена (c.3830G>T, p.Gly1277Val) в гене митоген-активированной протеинкиназы 8 — MAPK8IP3 (NM 001318852.2) у пациентки с отягощенным по материнской линии наследственным анамнезом. Ген локализован на коротком плече 16 хромосомы (16р13), характеризуется аутосомно — доминантным типом наследования. Продуктом экспрессии гена является белок JIP3, один из компонентов MAPK — сигнального пути, который участвует в аксональном транспорте (связывает биологически активное вещество с транспортными белками), а также в эмбриогенезе таламуса и гиппокампа. Гетерозиготные мутации ассоциированы с различными вариантами нарушений развития нервной системы (задержка психомоторного развития, нарушения интеллекта в 50% случаев сочетающиеся с аномалиями головного мозга). В 2019 г. S. Iwasawa и соавт. опубликовали серию клинических наблюдений, где из пяти пациентов с патологией ЦНС в двух случаях диагностировано гонадотропинзависимое преждевременное половое созревание. У всех пациентов идентифицированы гетерозиготные варианты в MAPK8IP3 [30]. Идентифицированная нами замена не описана ранее. Компьютерные алгоритмы предсказательности указывают нейтральный эффект варианта на функцию белка. Однако учитывая влияние гена на процессы пренатальной дифференцировки ряда структур головного мозга, а также на биологические процессы в нервных клетках нельзя исключить его влияние и взаимодействие с нейропептидами, опосредующими деятельность ГнРГ — регулятора, что требует дальнейших функциональных исследований (in vivo или in vitro).

Следующей находкой стало обнаружение ранее не описанного, гетерозиготного варианта (c.370A>G, p.Met124Val) в гене POU1F1 (NM 000306.4) у пациентки с отягощенным семейным анамнезом по материнской линии. Ген картирован на коротком плече 3 хромосомы (3р11.2), характеризуясь как аутосомно-рецессивным, так и аутосомно-доминантным типом наследования. Подавляющее большинство клинических фенотипов, обусловленных патогенными вариантами POU1F1, характеризуются изолированным и/ или комбинированным дефицитом гормонов аденогипофиза. В 2018 г. Firdevs Bas и соавт. опубликовали результаты собственных наблюдений за пациентами с комбинированным дефицитом тропных гормонов в сочетании с гонадотропинзависимым преждевременным половым созреванием [33]. Согласно литературным данным, взаимодействие POU1F1 с рядом транскрипционных факторов, в частности, c GATA — связывающим фактором 2, необходимо для целенаправленной и селективной дифференцировки тиреотропной и гонадотропной клеточных линий аденогипофиза [31]. Кроме того, в исследованиях in vivo продемонстрировано влияние POU1F1 на эволюцию и регуляцию активности рецептора ГнРГ [32]. Сверхэкспрессия GATA2 вероятная при мутациях POU1F1 приводит к увеличению гонадотропной дифференцировки и избыточной секреции гонадотропинов, что гипотетически объясняет развитие ППР [33]. Анализ in silico предсказывает нейтральный эффект замены на функцию белка. Для изучения роли мутации в патогенезе преждевременной активации ГГГО необходимы дальнейшие исследования, в частности, анализ наследования варианта для установления его косегрегации с болезнью и проверка патогенности функциональными исследованиями.

Не менее интересным стала идентификация нуклеотидного варианта c.452A>G, p.Glu151Gly в гене нейропептидного рецептора NPFFR1 (NM 022146.5). Ген локализован на длинном плече 10 хромосомы (10q22.1). Продуктом его экспрессии является нейропептидный рецептор FF1 (NPFFR1), который локализован на ГнРГ — секретирующих нейронах гипоталамуса. Среди афферентов к ГнРГ — секретирующим нейронам идентифицирована большая группа так называемых RF — амидов (аргинин — фенилаланиновые амиды), которые обладают стимулирующими или ингибирующими эффектами на ГнРГ — секретирующие нейроны, в частности, кисспептин относится к группе стимулирующих нейропептидов. Ингибирующую роль на секрецию ГнРГ оказывают RFRP — пептиды (аргинин — фенилаланин связанные пептиды), синтезируемые в ядрах гипоталамуса. При взаимодействии данных пептидов с соответствующими рецепторами (NPFFR1) реализуется их ингибирующий эффект на секрецию ГнРГ [34–36], что позволяет предположить их непосредственную роль в контроле ГГГО. В 2017 г. He Y и соавт. выдвинули гипотезу, согласно которой снижение уровня RFRP — пептидов и их ингибирующего эффекта на деятельность ГнРГ — нейронов гипоталамуса являются одним из механизмов запуска полового созревания. Аберрации в гене NPFFR1 могут быть причиной преждевременного снижения уровня последних, способствуя преждевременной активации ГГГО [37].

Таким образом, у 23% пациентов с ППР выявлены замены в генах, ассоциированных с нейроонтогенезом, а также с нейроэндокринными механизмами регуляции гипоталамо-гипофизарной оси. Идентифицированные замены не описаны ранее, биоинформатические алгоритмы предсказывают нейтральный эффект вариантов на функцию белка. Однако в связи с недостаточным количеством имеющихся данных невозможно исключить их влияние на регуляцию нейроэндокринных сигнальных путей и в патогенезе ППР, что диктует необходимость дальнейших исследований.

Ограничения исследования.

В ходе исследования могли возникнуть смешения результатов по причине недостаточного объема выборки, неопределенность оценок также связана с малым размером исследуемой выборки.

Направления дальнейших исследований

В продолжение работы планируются расширение выборки, дальнейшее проведение молекулярно-генетического исследования генов-кандидатов, имеющих взаимосвязь с развитием ППР центрального генеза. Следующим этапом планируется провести поиск взаимосвязи между клиническими данными и результатами молекулярно-генетического исследования в исследуемых группах.

## ЗАКЛЮЧЕНИЕ

Имеющиеся исследования по генам, ассоциированным с развитием гонадотропинзависимого преждевременного полового созревания, а также наличие данных о характере наследования и клинических проявлениях моногенных форм патологии, позволят улучшить эффективность молекулярно-генетического анализа и упростить диагностику наследственных форм заболевания. Проведенное нами исследование демонстрирует важность детального сбора наследственного анамнеза у детей с ППР для определения показаний к проведению молекулярно-генетического анализа.

Эффективность генетического тестирования возможно оценить по ранней постановке диагноза и снижению неблагоприятных последствий несвоевременно диагностированной патологии. Помимо понимания фундаментальных патогенетических механизмов развития заболевания, неотъемлемой частью исследования является медико-генетическое консультирование семей, необходимое для своевременного обследования и назначения патогенетической терапии.

## ДОПОЛНИТЕЛЬНАЯ ИНФОРМАЦИЯ

Источники финансирования. Исследование было проведено при содействии Фонда поддержки и развития филантропии «КАФ», бюджетных средств лечебно-профилактического учреждения — участника исследования (ФГБУ «НМИЦ эндокринологии» Минздрава России).

Конфликт интересов. Авторы декларируют отсутствие явных и потенциальных конфликтов интересов, связанных с содержанием настоящей публикации.

Благодарности. Авторы выражают благодарность Фонду поддержки и развития филантропии «КАФ» за помощь в проведении генетического исследования.

Участие авторов. Хабибуллина Д.А., Колодкина А.А. — концепция и дизайн исследования, предоставление материалов исследования и анализ данных, интерпретация результатов, подготовка финальной версии статьи; Визеров Т.В. — анализ данных, интерпретация результатов; Зубкова Н.А. — анализ полученных данных, написание статьи; Безлепкина О.Б. — редактирование текста, внесение ценных замечаний. Все авторы одобрили финальную версию статьи перед публикацией, выразили согласие нести ответственность за все аспекты работы, подразумевающую надлежащее изучение и решение вопросов, связанных с точностью или добросовестностью любой части работы.
